# A Cross-Sectional Study on Neuropathic Pain Associated With Quality of Sleep in Spondylosis Patients

**DOI:** 10.7759/cureus.59242

**Published:** 2024-04-28

**Authors:** Subhankar Das, Rajveer Singh, Utkarsh Agrawal, Somnath Nishad, Gyan Ranjan, Faiqua Jamal

**Affiliations:** 1 Pharmacy, School of Pharmaceutical Sciences, Jaipur National University, Jaipur, IND; 2 Orthopaedics, Jaipur National University Institute for Medical Sciences and Research Centre, Jaipur, IND

**Keywords:** spondylosis, degenerative changes, spine, quality of sleep, neuropathic pain

## Abstract

Introduction: Neuropathic pain (NP) is common in spondylosis patients. Cervical and lumbar spondylosis are more common in the elderly population. Spondylosis patients also suffer from poor quality of sleep (QOS). This study aims to find a correlation between NP and QOS in spondylosis patients.

Methods: We conducted a cross-sectional study and analyzed data using the chi-square test to correlate the NP with QOS. The Pittsburgh Sleep Quality Index (PSQI) and the Leeds Assessment of Neuropathic Symptoms and Signs, Self-complete (S-LANSS) questionnaires were used to assess QOS and evaluate neuropathic pain, respectively. Spondylosis was diagnosed based on the history, clinical examination, and radiological findings.

Results: A total of 72 spondylosis patients, with a mean age of 47.35 years, were included in this study. Out of 72 subjects, 52 (72.2%) patients had neuropathic pain (NP group), and 20 (27.8%) patients had non-neuropathic pain (non-NP group). In the NP group, 41 patients (78.8%) had poor QOS, while 11 (21.2%) had good QOS. In the non-NP group, eight (40%) had poor QOS, and 12 (60%) had good QOS.

Conclusion: This study concludes that neuropathic pain is associated with poor quality of sleep in spondylosis patients.

## Introduction

Neuropathic pain (NP) was defined in 1994 by the International Association for the Study of Pain (IASP). Neuropathic pain is characterized by the combination of a relatively small number of “core” pain qualities (particularly burning pain, electric shock-like pain, and dysesthesia) [1]. The major causes of neuropathic pain include diabetes, spinal cord injury, nerve injuries, shingles, cancer, stroke, and multiple sclerosis, and some common conditions are lumbar and cervical radiculopathy [2]. In the past 15 years, several clinical instruments have been developed in the form of questionnaires like S-LANSS (the Leeds Assessment of Neuropathic Symptoms and Signs, self-complete) painDetect and Douleur Neuropathique en 4 Questions (DN4), which use standard verbal pain descriptors like tingling, hot sensation, brushing, itching, and color change of pain site [3-5]. It is very difficult to treat neuropathic pain due to a lack of validated diagnostic parameters and operative instruments [6-7].

Spondylosis refers to age-related degenerative changes in the spine. These changes include disc desiccation, facet joint arthritis, osteophyte formation, and ligamentum flavum hypertrophy. Spondylotic changes in the cervical and lumbar spine can cause the narrowing of the spinal canal, leading to neck pain, back pain, or neuropathic pain such as tingling, numbness, and burning sensations in the arms or legs [8,9].

Sleep is essential for human functioning on a bio-psycho-social and cultural level, directly impacting their health and quality of life. Poor quality of sleep (QOS) leads to neurocognitive and physiological disorders, decreases metabolic rate, increases blood pressure, increases cortisol secretion, and increases insulin resistance [10]. It has already been proven that sleep helps to restore energy and concentration and has positive effects on every organ, such as the brain, heart, gastrointestinal system, etc. [11]. Patients with sleep disorders respond better to pharmacological treatment than to non-pharmacological treatment [12]. The Pittsburgh Sleep Quality Index (PSQI) questionnaire helps to detect the quality of sleep and may also be helpful in research on the relationships between sleep quality and other factors [13]. A direct relationship between neuropathic pain due to various causes and sleep quality has already been examined [14]. The aim of this study is to find out if neuropathic pain is associated with the quality of sleep in spondylosis patients.

## Materials and methods

A cross-sectional study was conducted at the Jaipur National University Institute for Medical Sciences and Research Centre (JNUIMSRC), Jagatpura, Jaipur, after obtaining approval from the Institutional Ethics Committee of JNUIMSRC. The IEC clearance number is JNUIMSRC/IEC/2022/100; date: December 30, 2022. The sample size of this cross-sectional study was 72. Patients diagnosed with cervical and lumbar spondylosis, age ≥18 years, and who provided consent and cooperation to participate in the research were included in the study. The study excluded chronic smokers, alcoholics, patients on sedative and antipsychotic medicines, diabetic patients, and those unwilling to participate.

Subjective sleep quality was assessed using the PSQI questionnaire. There were seven latency domains on this scale, i.e., length, quality, effectiveness of sleep patterns, sleep disorders, use of sleep-inducing drugs, overall sleep quality, and disruption of body processes during daytime sleepiness. Each of the seven items in the PSQI has a score from 0 to 3. The total range of this scale is 0 to 21 [15]. We categorized sleep quality as good when the overall value (global score) was less than five, and as poor when it was five or more [16]. The S-LANSS scale might be employed as a credible research tool where values under 12 were categorized as non-neuropathic pain, whereas values of 12 or above were categorized as neuropathic pain. The results were converted to a yes/no dichotomy [17]. In this study, spondylosis was diagnosed based on history, clinical examination, and radiological findings. Statistical analysis was done using SPSS 21.0 for Windows (IBM Corp., Armonk, NY). A Pearson chi-square test was used to determine the correlation or characteristics of NP with QOS in spondylosis patients.

Statistical analysis

Qualitative data were presented in numbers, and the study result was based on statistical analysis using the Pearson chi-square test. If the normality test failed, then a non-parametric test was used. The relationship between qualitative data was assessed by the chi-square test. A p-value of <0.05 was considered statistically significant. The data were entered in an MS Excel spreadsheet, and analysis was done using IBM SPSS Statistics for Windows, version 21.0 (released 2012; IBM Corp., Armonk, New York, USA).

## Results

This study included a total of 72 patients with either cervical or lumbar spondylosis. There were 29 (40.3%) female and 43 (59.7%) male patients. The mean age of the study was 47.35 years. Based on their educational background, 20.8% were university students, 18.1% had intermediate schools, 16% had secondary school students, and 38.9% were uneducated. The study included 33.3% farmers, 37.6% laborers, 20.8% office job holders, and 8.3% students. Most of the patients (43%) have family incomes of 20,000-30,000 INR per month (Table 1).

**Table 1:** Socio-demographic characteristics of spondylosis patients (n=72) INR: Indian Rupee

Variable	Categories	Frequency	Percentage (%)
Gender	Male	43	59.7
	Female	29	40.3
Educational level	University	15	20.8
	Intermediate	13	18.1
	Secondary	16	22.2
	Uneducated	28	38.9
Occupation	Farmer	24	33.3
	Laborer	27	37.6
	Office job	15	20.8
	Student	6	8.3
Family income per month (INR)	˂20,000	19	26.4
	20,000–30,000	31	43
	˃30,000	22	30.6

Out of 72 subjects, 52 (72.2%) patients had neuropathic pain (NP group), and 20 (27.8%) individuals had non-neuropathic pain (non-NP group). In the NP group, 41 patients (78.8%) had poor QOS, and 11 (21.2%) had good QOS. In the non-NP group, eight patients (40%) had poor QOS, while 12 patients (60%) had good QOS. In this study group, lumbar spondylosis was more common than cervical spondylosis. Out of 62 lumbar spondylosis patients, 36 were males and 26 were females, and out of 10 cervical spondylosis patients, four were males and six were females (Table 2).

**Table 2:** The relationship between pain and quality of sleep

Group	Patients	Good sleep	Poor sleep	P-value
NP	52 (72.2%)	11 (21.2%)	41 (78.8%)	0.002
Non-NP	20 (27.8%)	12 (60%)	8 (40%)	

**Figure 1:**
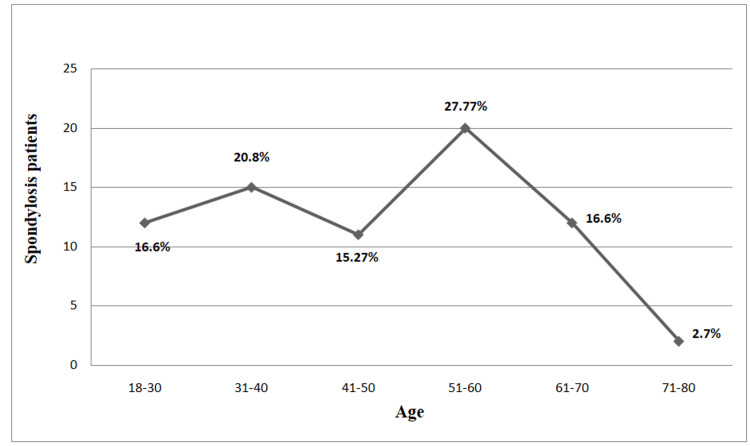
Age specific prevalence of spondylosis patients

During the research, the focus was on the prevalence of spondylosis patients in different age groups. It has been shown that those in the 51-60 age group are more prone to spondylosis than other age groups (Figure 1).

Pearson's chi-square test revealed a significance value of p=0.002. This p-value is between the NP group and the non-NP group regarding the quality of sleep. Spearman’s correlation study revealed the correlation coefficient (r) is −0.37. It indicates a moderate association between neuropathic pain and quality of sleep. Hence, from these findings, it has been proven that with an increase in neuropathic pain, there is a decrease in the quality of sleep.

## Discussion

In this study, it was observed that neuropathic pain is common in spondylosis patients, and out of 52 patients with neuropathic pain, 41 patients suffered from sleep disturbances leading to poor sleep quality. An association between neuropathic pain and poor sleep quality was observed. Similar outcomes have been documented by Zhou et al. [18]. Most of the studies have already proved that the relationship between sleep and neuropathic pain is bidirectional, with chronic pain strongly linked to disturbed sleep [19]. This bidirectional link is involved in common areas of the brain, particularly both the frontal cortex and the hippocampus, as a result of sleep deprivation [20].

Our study has been described in different ways based on spondylosis patients. The age of 18-80-year-old spondylosis patients was considered; 78.8% of patients had developed poor QOS with NP. Similar to the previous study, which considered 18-64-year-old patients, 46.2% had poor QOS with Melikoglu and Celik [21]. The presence of chronic pain, such as neuropathic pain, can significantly contribute to poor sleep quality and disrupt regular sleeping patterns; the quality of life of these patients is impaired by chronic sleep disorders, including insomnia and sleep apnea [22]. A prospective study by Finan et al. reported that the risk of chronic pain increases with sleep disturbance in spondylosis patients, increases the risk of chronic musculoskeletal pain and headache, and adversely impacts daily fluctuations in pain [23].

During this study, it was observed that lumbar spondylosis in male patients is more common than in female patients. A large population study in Saudi Arabia revealed that the most common person affected by lumbar spondylosis (53.1%). There is little evidence of an association between age and gender with lumbar and cervical spine disorders [24]. In a retrospective study, there was a significant difference between males and females in the incidence of lumbar spondylolysis. There was a higher prevalence of lumbar spondylosis, bone marrow edema, and L5 lesions in male patients than in female patients. 65% of males and 45% of females suffered lumbar spondylolysis; it was more common in males than in females [25].

Another study found a relationship between pain intensity and sleep quality. The intensity of chronic low back pain is increased in patients with poor sleep quality [26]. Bintang et al. [27] found a significant relationship between sleep quality and pain intensity (p=0.017). This is similar to our study, which found a significant relationship between NP and QOS (p=0.002).

The limitations of our study were the small sample size and the short period of time. In this study, no laboratory tests were performed to confirm whether neuropathic pain existed. Accordingly, NP and QOS were determined based on the scores of the S-LANSS and PSQI questionnaires. Future research should involve a cohort study with a large sample size and precise measurements of the factors influencing neuropathic pain and sleep quality.

## Conclusions

This study concluded that neuropathic pain is correlated with the quality of sleep in spondylosis patients. Males are more likely to develop lumbar spondylosis than women, and females are more likely to develop cervical spondylosis than males. Patients in the 51-60 age group are more likely to develop spondylosis and neuropathic pain.
